# Defining ovine dermal papilla cell markers and identifying key signaling pathways regulating its intrinsic properties

**DOI:** 10.3389/fvets.2023.1127501

**Published:** 2023-02-27

**Authors:** Shanhe Wang, Tingyan Hu, Mingliang He, Yifei Gu, Xiukai Cao, Zehu Yuan, Xiaoyang Lv, Tesfaye Getachew, Kai Quan, Wei Sun

**Affiliations:** ^1^College of Animal Science and Technology, Yangzhou University, Yangzhou, China; ^2^Joint International Research Laboratory of Agriculture and Agri-Product Safety of Ministry of Education of China, Yangzhou University, Yangzhou, China; ^3^International Joint Research Laboratory in Universities of Jiangsu Province of China for Domestic Animal Germplasm Resources and Genetic Improvement, Yangzhou University, Yangzhou, China; ^4^International Centre for Agricultural Research in the Dry Areas, Addis Ababa, Ethiopia; ^5^College of Animal Science and Technology, Henan University of Animal Husbandry and Economy, Zhengzhou, China; ^6^“Innovative China” “Belt and Road” International Agricultural Technology Innovation Institute for Evaluation, Protection, and Improvement on Sheep Genetic Resource, Yangzhou, China

**Keywords:** dermal papilla cell, Wnt/β-catenin, marker gene, hair-inducing activity, SOX18, PDGFRA

## Abstract

Dermal papilla cell (DPC), one of the key cell types during hair follicle development and regeneration, specifies hair size, shape and cycling. It is also an important *in vitro* screening model for hair growth. Although some characteristics of DPCs, such as agglutinative growth and marker genes, have been studied in mice and humans, the intrinsic properties of ovine DPCs and the regulatory mechanism of the intrinsic properties during continued culture *in vitro* remained unknown. In this study, based on our previous single-cell transcriptome sequencing on sheep lambskin, we verified *SOX18* and *PDGFRA* as the novel marker genes of ovine DPCs through immunofluorescence staining on skin sections and cultured DPCs. Using continued cell culture and alkaline phosphatase staining, we found that different from mice and humans, ovine DPCs exhibit particularly robust and stable aggregation with unbated alkaline phosphatase activity till 30 passages during continued culture *in vitro*. Also, we found that the expression of some marker genes and the activity of Wnt/β-catenin signaling differ between early passaged DPCs and multiple passaged DPCs. Further, using Wnt/β-catenin agonist and antagonist, we demonstrated that Wnt/β-catenin signaling could regulate cell aggregation and alkaline phosphatase activity of ovine DPCs through regulating FGF and IGF signaling. This study provides the basis for isolating ovine DPCs and defines their intrinsic properties, which contribute to improving wool performance and medicine of hair regeneration.

## 1. Introduction

Wool serves important physiological and social functions, and is also the raw material for textiles (1). It is produced from the hair follicle, which has a complicated structure and various cell types, one of the most important is dermal papilla cells (DPCs) (2, 3). DPCs are derived from dermal condensate after interacting with ectodermal cells during hair follicle morphogenesis in the embryonic period (4, 5). After follicle morphogenesis, the hair follicle undergoes cyclic transformation postnatally (6). The DPCs are the signal center for regulating hair follicle morphogenesis and cycle through regulating cell proliferation and differentiation of hair follicle stem cells and matrix cells (7, 8). Previous research showed that the hair follicle would stop growing after excising the dermal papilla from the hair follicle (9, 10), while functional and intact hair follicles could be successfully reconstructed *in vivo* using original or early passaged DPCs and hair follicle stem cells (11, 12). In addition, studies have shown that DPCs determine hair follicle type, hair growth and hair quality (13–15).

As the critical function of DPC, scientists have been trying to clarify its characteristics. At the earliest, researchers isolated and cultured DPCs through enzyme-assisted microdissection (16). After that, some marker genes of murine DPCs were identified through the transcriptome sequencing (17, 18). Based on known marker genes, purer DPCs were isolated and novel marker genes were identified in mice and humans through fluorescence labeling and fluorescence-activated cell sorting (19, 20), making a big push for investigating the molecular mechanism governing hair follicle development. Recently, the dynamic transcriptional map and cell heterogeneity were revealed by the single-cell transcriptome sequencing (21–23). However, the molecular characteristics of DPCs are specific to species, and the molecular characteristics of ovine DPCs still remain unknown.

DPCs cultured *in vitro* is an essential part of grafted cells for reconstituting hair follicle *in vivo* (24, 25), and is an important *in vitro* screening model for hair growth (26). However, DPCs cultured *in vitro* usually lose hair-inducing activity (27), which is a bottleneck for investigating DPCs and hair follicle development. Research showed that early passaged DPCs exhibit a multilayer aggregative growth character and express marker genes such as *ALPL*, while the passaged DPCs with more than 6 passages will lose these characteristics, which is consistent with the inability to induce hair regeneration (28, 29). Therefore, clarifying the molecular mechanism of losing the intrinsic properties of passaged DPCs may have important implications for hair regeneration and hair cycle. A previous study had demonstrated that the passaged DPCs could maintain the hair-inducing activity by co-culturing with cells secreting Wnt3a or conditioned medium (28), which indicated that Wnt/β-catenin signaling plays an important role in maintaining the hair-inducing activity of DPCs. In addition, other research showed that supplementation of FGF2 or 3-D culture would restore the hair-inducing activity of DPCs (30, 31).

Previously, we identified the marker genes of ovine DPCs using single-cell transcriptome sequencing (32). However, the validity and the conservation among different species as well as the associated relationship of these marker genes and the intrinsic properties of DPCs are still elusive. On the base of the previous research, we verified the marker genes through immunofluorescence staining on skin sections and cultured DPCs. Meanwhile, we detected the difference in intrinsic properties and gene expression between early passaged and multiple passaged DPCs. Furtherly, we clarified the potential molecular mechanism of maintaining hair-inducing activity. This study provides the basis for isolating DPCs and clarifies their intrinsic properties, which will contribute to improving wool performance and medicine of hair regeneration.

## 2. Results

### 2.1. Identifying the marker genes of ovine DPCs

**Figure 1:**
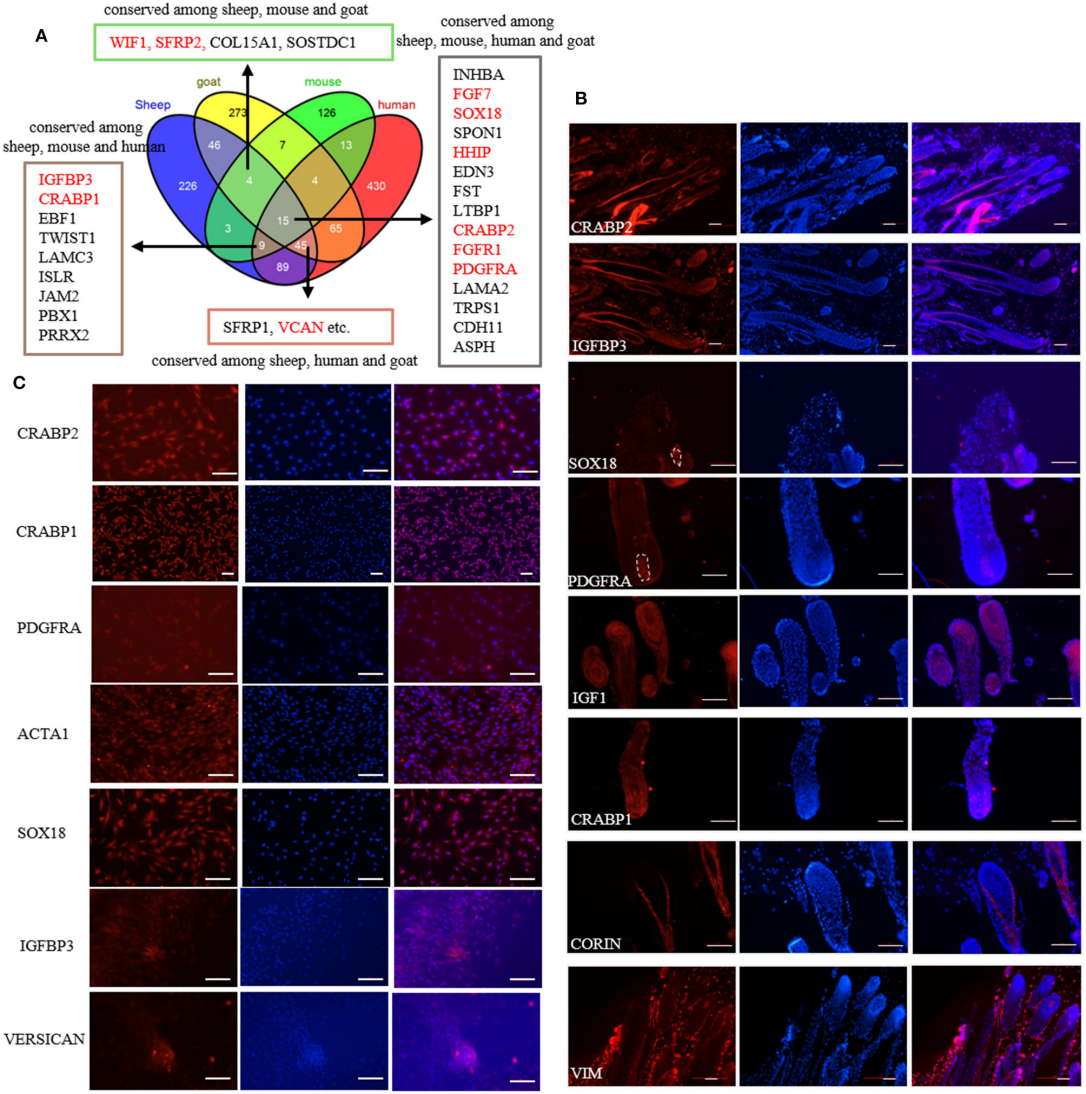
Identification of marker genes of ovine DPCs. **(A)** Comparative analysis of dermal papilla cell signature genes from different species. The genes listed in the textbox are conserved among sheep and other two or three species. The genes marked in red were furtherly verified in the subsequent study. **(B)** Immunofluorescence staining of potential markers genes in skin paraffin section. **(C)** Immunofluorescence staining of potential markers genes in cultured ovine DPCs. Red fluorescence indicated the expression pattern of the interested protein. Nucleus was stained with Hoechst in blue. Scale bars, 50 μm.

Previously, we screened the signature genes of ovine DPCs using single-cell transcriptome sequencing (32). However, the conservation and the validity of these marker genes are still elusive. To understand the conservation of these marker genes, we compared the transcriptome data of DPCs from different species. As a result, 15 marker genes are conserved among sheep, mice, humans and goats, 73 marker genes are conserved among sheep and other two species, while 226 genes are specific for sheep (Figure 1A; Supplementary Table S2). Subsequently, we verified some conserved marker genes through immunofluorescence staining on sheep skin paraffin sections and cultured DPCs. The results showed that CRABP2 was expressed in ovine DP and outer root sheath, IGFBP3 was expressed in DP, matrix and outer root sheath, CRABP1 and IGF1 were expressed in DP and matrix, SOX18 and PDGFRA were specifically expressed in DP, while VIM was specifically expressed in dermal cells as a positive control (33). In addition, *CORIN*, a known marker gene in mice (34) while not screened in sheep, expressed in dermal sheath rather than DP (Figure 1B), *SOX2* and *CD133*, the well-known marker genes in mice (7), were not detected in sheep. Besides, we further verified the expression of these markers in cultured DPCs through immunofluorescence. The results showed that CRABP1, CRABP2, PDGFRA and α-SMA were widely expressed in cultured DPCs, in which α-SMA is a positive control as a certified marker (35). IGFBP3 was highly expressed in agglutinative cells, which was similar to VERSICAN (Figure 1C). It is worth noting that PDGFRA was expressed in the cell membrane, which could be used as the cell surface marker. These results together implied that *SOX18* and *PDGFRA* were novel marker genes of ovine DPCs and *IGFBP3* was a novel marker gene for aggregated DPCs.

### 2.2. The change of intrinsic properties of DPCs during a period of culture *ex vivo*

**Figure 2:**
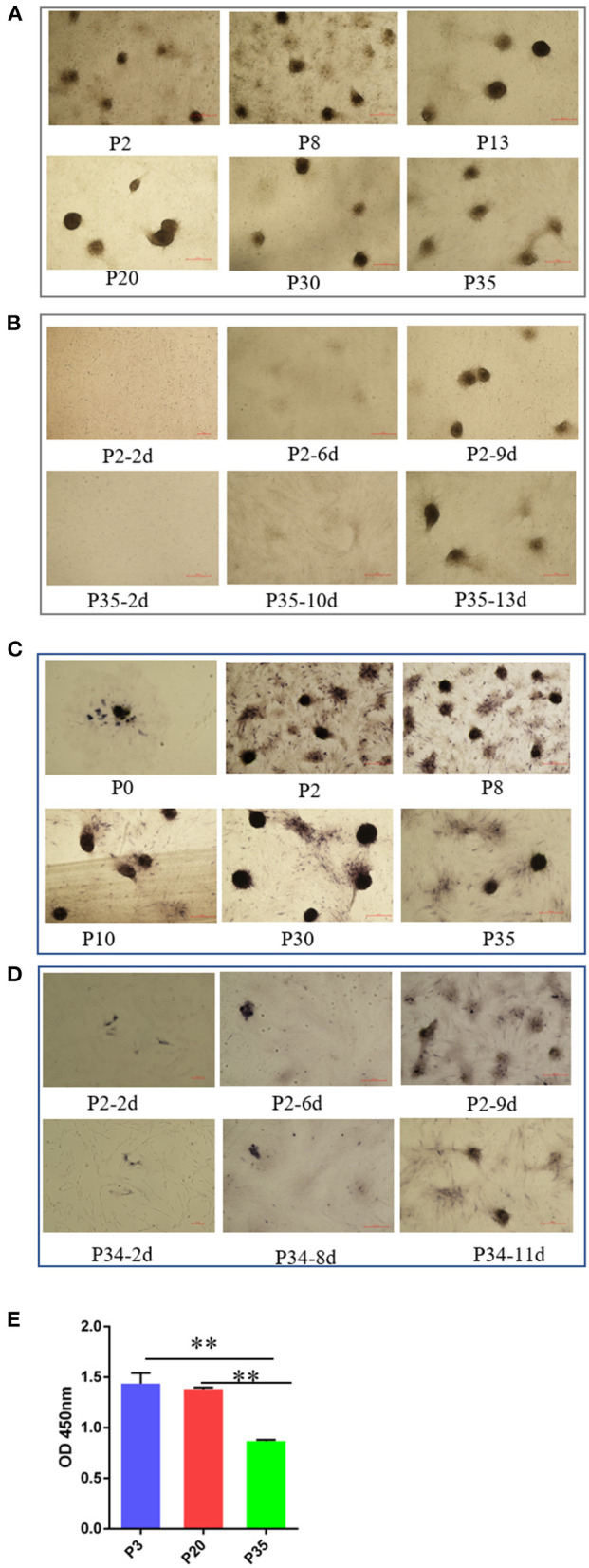
The change of intrinsic properties of DPCs during a period of culture *ex vivo*. **(A)** The ability of cell aggregation from early passaged ovine DPCs to multiple passaged DPCs. The agglutinated cells will eventually form three-dimensional spheroids projecting up from the culture substrate **(B)**. The difference of cell aggregation ability between early passaged ovine DPCs to multiple passaged DPCs. **(C)** Alkaline phosphatase activity from early passaged ovine DPCs to multiple passaged DPCs. The cells positive for alkaline phosphatase showed a dark brown color. **(D)** The difference of alkaline phosphatase activity among DPCs before and after cell aggregation. **(E)** The cell viability among different passages' DPCs. **P < 0.01.

Cell aggregation is a critical characteristic of DPCs, which is typically lost after a period of culture *ex vivo* in humans and mice (27). In this study, we detected the ability of cell aggregation from early passaged ovine DPCs to multiple passaged DPCs. In contrast, we found ovine DPCs consistently aggregated for at least 35 passages, in which the aggregated cells will eventually form three-dimensional spheroids projecting up from the culture substrate (Figure 2A). For those cells < 30 passages, the DPCs presented obvious aggregation after about 7 days, However, for those cells with more than 30 passages, they took a long time to form aggregation (Figure 2B). Although, the aggregate size had no significant difference between early passaged DPCs and multiple passaged DPCs. These results suggested that ovine DPCs exhibit particularly robust and stable aggregation, despite the ability of cell aggregation being decreased till 30 passages.

Alkaline phosphatase is expressed in DP *in vivo* and is a marker of follicle-inducing activity (19). Previous study demonstrated that early passaged DPCs have alkaline phosphatase activity, while the alkaline phosphatase activity will lose after a period of culture (36). In contrast, we found ovine DPCs have alkaline phosphatase activity from early passaged ovine DPCs to multiple passaged DPCs (Figure 2C). It should be noted that alkaline phosphatase staining was observed in most ovine DPCs composing aggregates, while was observed in a small portion of DPCs before confluence (Figure 2D). These results suggested that the process of cell agglutination may affect alkaline phosphatase activity.

In addition, the cell viability results showed that there was no significant difference between P3 and P20 of DPCs, while the cell viability significantly decreased in P35 compared with P3 or P20 (*P* < 0.01) (Figure 2E). This might be a potential reason for the declined cell aggregation ability.

### 2.3. The expression changes of marker genes and Wnt signaling of DPCs during a period of culture *ex vivo*

**Figure 3:**
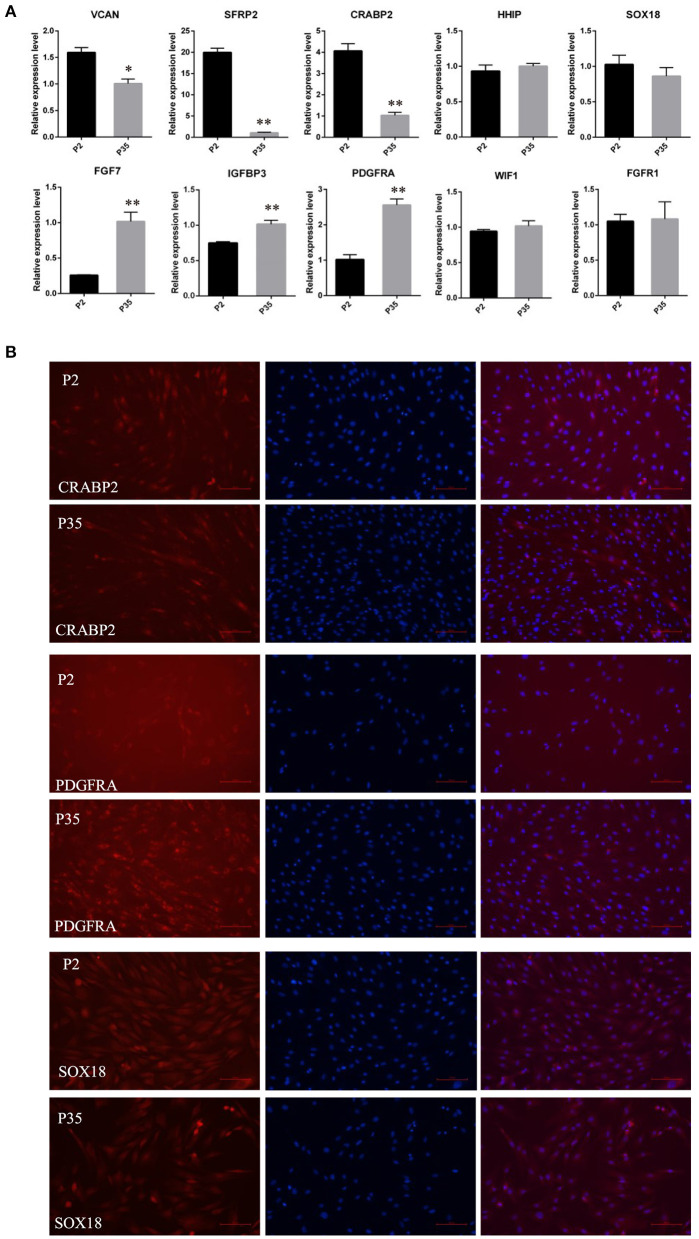
The expression changes of marker genes of DPCs during a period of culture *ex vivo*. **(A)** qRT-PCR of marker genes of ovine DPCs in passage 2 and passage 35. **(B)** Immunofluorescence staining of CRABP2, PDGFRA and SOX18 in early passaged ovine DPCs and multiple passaged DPCs. The expression of specific gene was quantified relative to the expression level of GAPDH using the comparative cycle threshold (ΔΔCT) method. The data were expressed as the mean ± SE (*n* = 3). *P < 0.05, **P < 0.01. Red fluorescence indicated the expression pattern of the interested protein. Nucleus was stained with Hoechst in blue. Scale bars, 100 μm.

Subsequently, we detected the expression changes of marker genes from early passaged ovine DPCs to multiple passaged DPCs using qPCR. As a result, the expression of *SOX18, HHIP, WIF1*, and *FGFR1* have no significant differences between early passaged ovine DPCs and multiple passaged DPCs. Of note, the expression of *CRABP2, SFRP2*, and *VCAN* were dramatically decreased in multiple passaged DPCs compared with early passaged ovine DPCs, while *IGFBP3, FGF7*, and *PDGFRA* were dramatically increased in multiple passaged DPCs compared with early passaged ovine DPCs (Figure 3A). Furtherly, immunofluorescence results of CRABP2, PDGFRA, and SOX18 confirmed the above results (Figure 3B).

**Figure 4:**
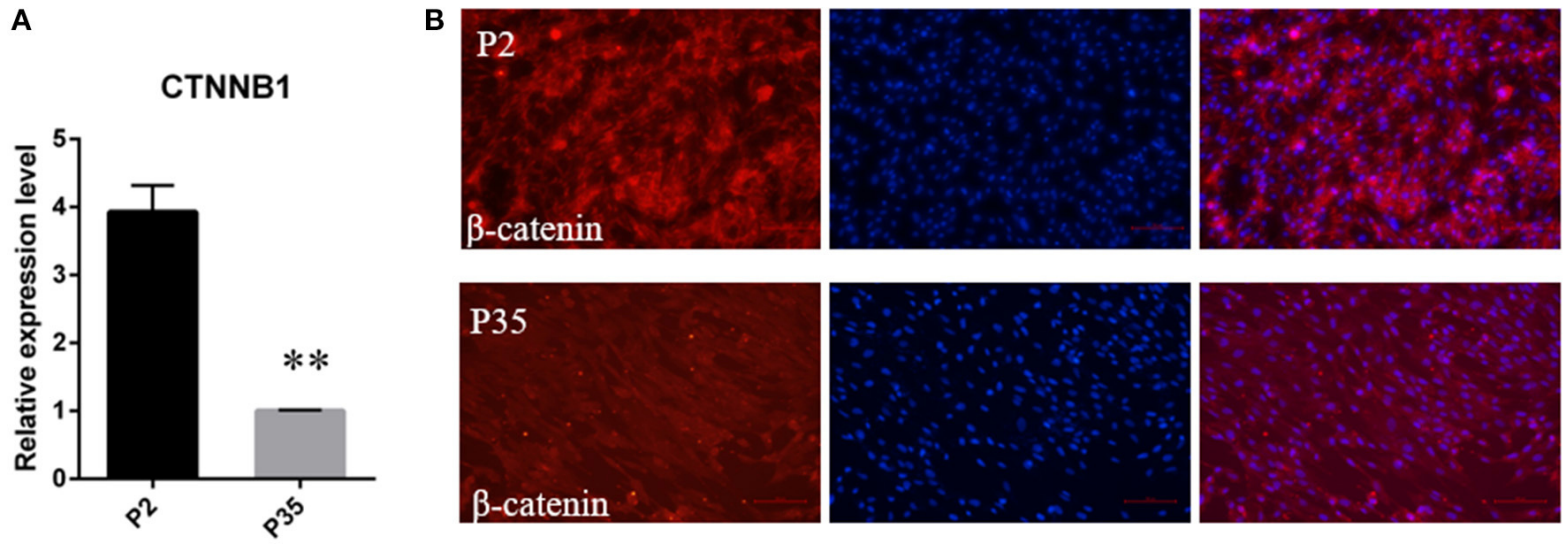
The change of Wnt/β-catenin signaling activity during a period of culture *ex vivo*. **(A)** qRT-PCR of CTNNB1, the transcriptional coactivator of Wnt signaling, in passage 2 and passage 35 DPCs. **(B)** Immunofluorescence Staining of β-catenin in early passaged ovine DPCs and multiple passaged DPCs. **P < 0.01.

Meanwhile, considering the important role of the Wnt signaling in hair follicle development and maintaining DPC hair-inducing activity (28), we further quantitated the mRNA expression level of *CTNNB1*, the transcriptional coactivator of Wnt signaling, using qPCR. The result showed that *CTNNB1* was downregulated in multiple passaged DPCs compared with early passaged ovine DPCs (Figure 4A). Additionally, with immunofluorescence, we found the cells positive for nuclear β-catenin decreased in multiple passaged DPCs compared with early passaged ovine DPCs, which suggested that Wnt signaling decreased after a period of culture *ex vivo* (Figure 4B).

### 2.4. The effect of Wnt signaling on the intrinsic properties of DPCs

**Figure 5:**
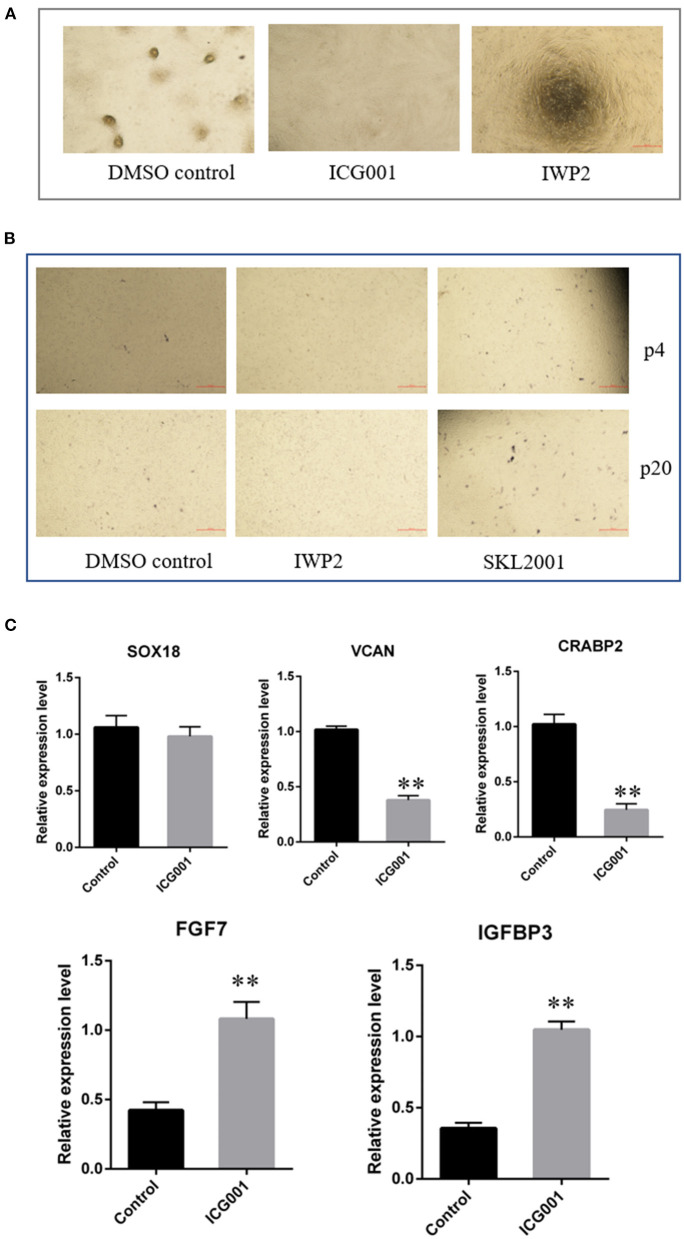
The effect of Wnt signaling on DPCs intrinsic properties. **(A)** The effect of Wnt signaling on cell aggregation of DPCs. **(B)** The effect of Wnt signaling on alkaline phosphatase activity of DPCs. **(C)** qRT-PCR of marker genes of ovine DPCs treated with Wnt/β-catenin antagonist ICG001. The data was expressed as the mean ± SE (*n* = 3). **P < 0.01.

As Wnt signaling decreased after a period of culture *ex vivo* accompanied by decreased cell aggregation ability, we speculated Wnt signaling plays an important role in maintaining the hair-inducing ability of ovine DPCs. To verify it, we detected the influence of Wnt signaling on the intrinsic properties of DPCs using Wnt/β-catenin agonist and antagonist. The Wnt/β-catenin agonist and antagonist worked well as verified by β-catenin immunofluorescence (Supplementary Figure S1). As a result, ovine DPCs lost the ability of cell aggregation when treated with Wnt/β-catenin antagonist ICG001 and IWP2 (Figure 5A). Meanwhile, the cells positive for alkaline phosphatase staining declined when treated with Wnt/β-catenin antagonist IWP2, while increased when treated with Wnt/β-catenin agonist SKL2001 (Figure 5B). Besides, gene expression analysis of some DP signature genes was conducted. The mRNA level *VCAN* and *CRABP2* were decreased in the DPCs treated with Wnt/β-catenin antagonist ICG001 compared with the control group. The mRNA level of *SOX18* had no significant difference between DPCs treated with Wnt/β-catenin antagonist ICG001 and the control group. It is worth noting that *FGF7*, an intercellular signaling factor known to act on follicular keratinocytes (37), was increased in the DPCs treated with Wnt/β-catenin antagonist. *IGFBP3* transcripts, which encode a secreted inhibitor of IGF signaling (38), were increased in the DPCs treated with Wnt/β-catenin antagonist (Figure 5C). These results suggested that Wnt/β-catenin plays an important role in maintaining the intrinsic properties of DPCs through regulating FGF and IGF signaling.

## 3. Discussion

Taking advantage of marker genes for specific cells, researchers could isolate the cells from heterogeneous cells through fluorescence-activated cell sorting (FACS) (39), and conduct the cell-specific transgenic animal models (40), which could greatly promote the research of this cell. Researchers have sorted the murine DPCs through FACS based on *CD133* and *SOX2* marker genes (19). However, at present, researchers isolated ovine DPCs still using the method of enzyme-assisted microdissection (41), due to lack of related marker genes, which was time-consuming and unpurified. In this study, we verified that *SOX18* and *PDGFRA* were novel marker genes of ovine DPCs, in which *PDGFRA* could be used for FACS as the cell surface marker. It should be noted that PDGFRA was also expressed in other dermal cells, FACS should conduct on single hair follicles after removing dermal sheath cells. Previous studies showed that mutations in *SOX18* underlie hair follicle defects in ragged mice and inhibit dermal papilla maturation and differentiation indicating the critical function of *SOX18* in DPCs (42, 43). *PDGFRA* was found as the dermal condensate marker (44), however its function in hair follicle development is still unknown. Meanwhile, consistent with our previous study (32), we found IGFBP3 was highly expressed in agglutinative cells, which indicated that IGFBP3 may play an important role in cell aggregation.

Since DPCs *in vivo* are enumerable and difficult to micro-dissect, scientists have been working on isolating and culturing DPCs with hair-inducing ability *in vitro*. However, in humans and mice, DPCs would lose the hair-inducing ability quickly after a period of culture *ex vivo*, in accordance with a decreased ability of cell aggregation, declined alkaline phosphatase activity and failure to induce hair regeneration *in vivo*, which restricts its application (45). In contrast, we found ovine DPCs exhibited particularly robust and stable aggregation even after multiple passages, which indicated that ovine DPCs could maintain hair-inducing ability well when cultured *in vitro*. We, therefore, speculated that ovine DPCs could act as a better *in vitro* screening model for hair growth research as its stable aggregation *in vitro* imperfectly replicates the dermal papilla physiological niche *in vivo*. In addition, ovine DPCs could be a better seed cell for hair regeneration study. We hypothesized that the different abilities to maintain cell aggregation between sheep and mice may be due to the different hair cycles and natural selection, in which sheep have been artificially selected for intensive wool growth with hair cycles of about 1 year (46), while mice have not been selected for hair with hair cycles about 21 days (6). Another significant finding is that alkaline phosphatase activity will increase during cell agglutination and have no difference between early passages and multiple passages, while previous studies in humans and mice showed that the alkaline phosphatase activity may decline after a period of culture and is a marker of follicle inducing activity (34).

The expression of some marker genes changed significantly between early passaged ovine DPCs and multiple passaged DPCs, which indicated these marker genes are associated with hair-inducing ability. We found Wnt/β-catenin signaling played an important role to regulate the intrinsic properties of ovine DPCs. Consistent with our research, both *in vivo* and *in vitro* studies in mice and humans have shown that Wnt/β-catenin signaling in DPCs is required for hair follicle-inductive properties (28, 47). Besides, Wnt signaling could maintain the hair-inducing activity of the cultured dermal papilla (48). In addition, we found IGF and FGF signaling were changed accompanied by the change of Wnt/β-catenin signaling. FGFs and IGFs are intercellular signaling factors known to act on DPCs themselves and follicular keratinocytes, which have been demonstrated to play an important role in regulating hair follicle development. Consistent with this, *in vitro* study using alternative culture conditions for DPCs reported decreased expression of Fgf7 when the Wnt/β-catenin pathway was activated (34), while deletion of β-catenin in the DP results in alterations of *IGFBPs* gene expression in the DP (49). These results suggested that Wnt/β-catenin signaling regulates signals from the DPCs that orchestrate the intrinsic properties of DPCs.

In summary, in this study we defined *SOX18* and *PDGFRA* as the novel marker genes of ovine DPCs. Ovine DPCs exhibit particularly robust and stable aggregation and unbated alkaline phosphatase activity during continued culture *in vitro*. Besides, some marker genes and key signaling pathways regulating its intrinsic properties were identified.

## 4. Materials and methods

### 4.1. Animals

Skin tissues of Hu sheep were obtained from Suzhou sheep breeding farm (Suzhou, Jiangsu, China) for isolating DPCs and making tissue sections. Briefly, three healthy lambs (3 days old, 1 male and 2 female) were anesthetic through hypodermic injection of 2% lidocaine hydrochloride (10 mg), then skin samples ~1 cm^2^ and 2 mm deep were harvested from the body side of lambs. The skin samples were divided into two parts and stored in Dulbecco's modified essential medium (DMEM)/F12 medium (Thermo Fisher Scientific, MA, USA) with penicillin-streptomycin (Thermo Fisher Scientific, MA, USA) or 4% paraformaldehyde (Solarbio, Beijing, China) for subsequent analysis. The wound was sutured. All the experimental procedures and sheep used in this study received prior approval from the Experimental Animal Management Committee of Yangzhou University (No: NSFC2020-NFY-1).

### 4.2. Comparative analysis of DPCs signature genes from different species

Previously, we had conducted single-cell transcriptome sequencing on Hu sheep lambskin from curly wool lambs and straight wool lambs (19). Based on other related published literature in humans, mice and cashmere goats (32, 33, 50), the transcriptome data of DPCs from different species were compared to analyze the conservation of marker genes in DPCs. Conserved marker genes among different species and sheep-specific marker genes were identified. The results were shown through a Venn diagram (https://bioinfogp.cnb.csic.es/tools/venny/index.html).

### 4.3. Immunofluorescence staining

Immunofluorescence staining on the skin paraffin section and cultured DPCs were performed as previously described (32, 51). Briefly, skin samples fixed with paraformaldehyde, as mentioned in Section 4.1, were dehydrated in gradient ethanol series. The samples were then rinsed with xylene for 30 min and further embedded in paraffin. Embedded samples were serially cut into longitudinal sections at 5 μm thickness using a microtome (Leica RM2255, Nussloch, Germany). Before staining, slides were deparaffinized in xylene for 30 min and then treated in 0.01 M sodium citrate buffer at 96°C (10 min) for antigen recovery. Then the slides were blocked using PBS supplemented with 10% goat serum and 3% bovine serum albumin (Merck KGaA, Darmstadt, Germany) at room temperature for 40 min. Ovine DPCs grown on coverslips were fixed with 4% paraformaldehyde for 15 min. They were then permeabilized in 0.5% Triton X-100 PBS solution for 15 min, and incubated in block solution (PBS with 0.5% triton X-100 and 10% goat serum) for >30 min at room temperature. Primary antibodies against target proteins were then incubated with the skin paraffin section or cultured DPCs samples at 4°C overnight. Fluorescently-labeled secondary antibodies were used to specifically bind to primary antibodies at 37°C for 30 min. The primary antibodies and secondary antibodies used in this study are listed in Table 1. Hoechst33342 (Beyotime Biotechnology, Shanghai, China) was used for nuclei staining and the slides were finally mounted with Vecatshield mounting media (Vector, USA). Each primary antibody was conducted on three biological replicates. Fluorescent pictures were taken under a Fluorescence Inversion Microscope System (Nikon, Tokyo, Japan).

**Table 1:** List of antibodies used for immunofluorescence staining.

**Antibody**	**Manufacture**	**Cat no**.
CRABP1 antibody	Sangon Biotech	D224674
CRABP2 antibody	Abcam	ab211927
IGFBP3 antibody	Santa Cruz	sc-374365
IGF1 antibody	Santa Cruz	sc-518040
SOX18 antibody	Affinity	DF8720
PDGFRA antibody	Abcam	ab203491
Anti-corin antibody	Abcam	ab255812
WIF1 antibody	Santa Cruz	sc-373780
SOX2 antibody	Santa Cruz	sc-365823
VIM antibody	Santa Cruz	sc-6260
Anti-ACTA1 rabbit polyclonal antibody	Sangon Biotech	D121592
β-catenin mouse polyclonal antibody	Proteintech	66379-1-lg
Anti-VCAN rabbit polyclonal antibody	Sangon Biotech	D223532
Goat anti-mouse IgG H&L (Alexa Fluor^®^ 555)	Abcam	Ab150114
Goat anti-rabbit IgG H&L (Alexa Fluor^®^ 555)	Abcam	Ab150078
Goat anti-rabbit IgG H&L (Alexa Fluor^®^ 488)	Abcam	Ab150077

### 4.4. DPCs isolation and culture

Primary DPCs were isolated *via* micro-dissecting dermal papillae and explanting them in a culture medium as previously described (32). The DPCs were cultured in adherent culture dishes with DMEM/F12 media supplemented with 10% fetal bovine serum (FBS, Thermo Fisher Scientific, MA, USA), penicillin (100 U/ml), and streptomycin (100 mg/ml) at 37°C with 5% CO_2_. DPCs migrated from original and compact DP at 3–4 days after seeding, which were named P0. Cells were passaged every 2 days after P1. DPCs are well known to aggregate in culture, which is likely to relate to hair-inducing activity (3). The DPCs with agglutinate capability will eventually form three-dimensional spheroids projecting up from the culture substrate. We detected DPC aggregation for different passaged DPCs. The images were acquired by Fluorescence Inversion Microscope System (Nikon, Tokyo, Japan).

### 4.5. Alkaline phosphatase staining

Before staining, the DPCs were fixed with 4% formaldehyde for 30 min. The alkaline phosphatase activity of DPCs was detected by using BCIP/NBT Alkaline Phosphatase Color Development Kit (Beyotime, Shanghai, China) following the manufacturer's instructions. Each group set 3 replicates. Finally, the images were acquired by Fluorescence Inversion Microscope System (Nikon, Tokyo, Japan).

### 4.6. Quantitative real-time PCR

Total RNA was extracted from DPCs using Trizol reagent (Thermo Fisher Scientific, MA, USA) following the manufacturer's instructions. The first-strand cDNA was obtained using a PrimeScript™ RT reagent Kit with gDNA Eraser (TAKARA, Beijing, China), and then was subjected to quantification of the mRNA with GAPDH as an endogenous control on the Bio-Rad CFX96 Touch™ Real-Time PCR Detection System (Bio-Rad, CA, USA). qRT-PCR reaction system and amplification procedure were performed as previously described (51). Each group included at least 3 samples, and all reactions were performed in triplicate for each sample. The primers and annealing temperatures for genes were listed in Supplementary Table S1.

### 4.7. Wnt/β-catenin agonist and antagonist

Wnt/β-catenin agonist SKL2001 (MedChemExpress, Shanghai, China) and antagonist IWP2 (Beyotime Biotechnology, Shanghai, China) as well as ICG001 (Beyotime Biotechnology, Shanghai, China) were used to study the effect of Wnt/β-catenin on DPCs. SKL2001 (52), a small molecule that controls the biological activity of Wnt, was added to DPCs (P4 and P20) at a final concentration of 20 μM based on the preliminary experiment. IWP-2 (53), an inhibitor of Wnt processing and secretion, was added to DPCs (P4 and P20) at a final concentration of 20 μM and ICG-001 (54), an inhibitor of β-catenin/TCF mediated transcription, was added to DPCs (P4 and P20) at a final concentration of 10 μM based on the preliminary experiment.

### 4.8. Statistical analysis

SPSS 20.0 was used for statistical analysis, the student's *t*-test was utilized to calculate the statistical significance between the two groups and One-Way ANOVA was used for many groups. Composite data are shown as the mean ± standard error. A *P*-value of ≤ 0.05 was considered significant.

## Data availability statement

The original contributions presented in the study are included in the article/Supplementary material, further inquiries can be directed to the corresponding author.

## Ethics statement

The animal study was reviewed and approved by Animal Care and Use Committee at Yangzhou University.

## Author contributions

Conceptualization: SW and WS. Methodology, formal analysis, and writing—original draft preparation: SW. Validation: SW, TH, MH, YG, ZY, and XC. Writing—review and editing: TG. Visualization: XL. Supervision: WS. Project administration: TG and WS. Funding acquisition: KQ and WS. All authors have read and agreed to the published version of the manuscript.
