# Supplementary material for: Defining ovine dermal papilla cell markers and identifying key signaling pathways regulating its intrinsic properties
Source: Front Vet Sci. 2023 Feb 27;10:1127501. doi: 10.3389/fvets.2023.1127501 (PMC10009177; doi:10.3389/fvets.2023.1127501)
Supplement: Supplementary file 1 [file Data_Sheet_1.ZIP › Supplementary Files/Figure S1.pdf]

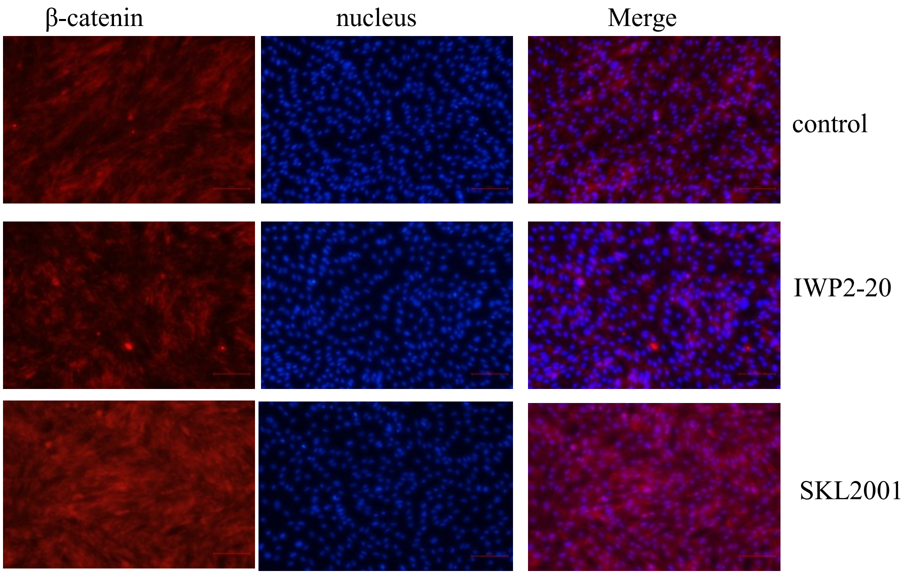

Figure S1. Immunofluorescence Staining of β-catenin in DPCs treated with Wnt/β-catenin agonist and antagonist. Fluorescence indicated the expression pattern of interest protein. Nucleus was stained with Hoechst in blue. Scale bars, 100 μm.
