# Supplementary material for: Defining ovine dermal papilla cell markers and identifying key signaling pathways regulating its intrinsic properties
Source: Front Vet Sci. 2023 Feb 27;10:1127501. doi: 10.3389/fvets.2023.1127501 (PMC10009177; doi:10.3389/fvets.2023.1127501)
Supplement: Supplementary file 1 [file Data_Sheet_1.ZIP › Supplementary Files/Table S1 Primer list for qRT-PCR .docx]

**Table S1** **Primer list for qRT-PCR**

| Gene | **Accession number** | **Forward primer** | **Reverse primer** |
| --- | --- | --- | --- |
| CRABP2 | XM_004002627.5 | ACAGGAGGGGGACACTTTCT | TTGGGACCCTCTCCCTTCAA |
| HHIP | XM_004017224 | GTGGCCTGTGCTTTCCTGAT | AGAATGAAGAGGCGGTGGGA |
| WIF1 | XM_004006493.5 | AAGCCTGTACCTGTGGATCG | CTGGCATTCTCTGCTGTGCT |
| PDGFRA | XM_004009830.5 | TTTGTGACGGTGCTGGAAGT | TCCTCCACGATGACCAAGGA |
| SFRP2 | NM_001163053.1 | TCCGAAGGTATGTGAAGCCTG | GGACACTCCGTTCAGCTTGT |
| FGF7 | XM_012180603.4 | ATCAGGACAGTGGCTGTTGG | TTTCTCCTCCGCTGTGTGTC |
| GAPDH | NM_001190390.1 | TCTCAAGGGCATTCTAGGCTAC | GCCGAATTCATTGTCGTACCAG |
| VCAN | XM_004009067.5 | TACAAAGGGAGGGTGTCGGT | AAGCCTTCTGTGCCATCTCA |
| FGFR1 | XM_027962632.2 | TTCGGATAAGGGCAACTACAC | AATCTTACTCCCGTTCACC |
| SOX18 | XM_027976914.2 | GCTTTGCGGCCTCTACTAC | GAACTCGGTCAGGTCCACGTC |
| IGFBP3 | NM_001159276.1 | AGTAGTCAGTGAAACGCTGTT | GCCTTGTCTCTCGTCTTACA |
